# Does external beam radiation boost to pelvic lymph nodes improve outcomes in patients with locally advanced cervical cancer?

**DOI:** 10.1186/s12885-019-5594-4

**Published:** 2019-04-25

**Authors:** Caryn Wujanto, Bok Ai Choo, David Tan, Arunachalam Ilancheran, Joseph Ng, Jeffrey J. H. Low, Liang Shen, Johann Tang, Vicky Koh

**Affiliations:** 1 Department of Radiation Oncology, National University Cancer Institute, National University Hospital, NUHS Tower Block Level 7, Singapore, 119228 Singapore; 2 Department of Medical Oncology, National University Cancer Institute, National University Hospital, NUHS Tower Block, Level 7, Singapore, 119228 Singapore; 3 Division of Gynaecologic Oncology, National University Cancer Institute, National University Hospital, NUHS Tower Block, Level 12, Singapore, 119228 Singapore; 4 Biostatistics Unit, Yong Loo Lin School of Medicine, National University of Singapore, 21 Lower Kent Ridge Road, Singapore, 119077 Singapore

**Keywords:** Cervical cancer, Radiotherapy, Pelvic node, Radiation boost

## Abstract

**Background:**

Current recommendation for locally advanced cervical cancer includes pelvic external beam radiation therapy (EBRT) with concurrent chemotherapy followed by brachytherapy. Involvement of pelvic lymph nodes is an important prognostic factor in locally advanced cervical cancer and recurrence commonly occurs despite definitive treatment. To date, there is no standard guideline on whether an EBRT boost should be applied to involved pelvic lymph nodes. Our study aims to assess if pelvic EBRT boost would reduce recurrence, benefit survival, and affect associated toxicities.

**Methods:**

We conducted a retrospective review of locally advanced cervical cancer cases treated with definitive treatment at our institution. Involvement of pelvic lymph nodes were assessed on CT, MRI (> 10 mm or suspicious features) or PET scan (SUVmax > 2.5). EBRT dose ranged from 45 to 50.4 Gy with nodal boost ranging from 3.6–19.8 Gy.

**Results:**

Between 2008 to 2015, 139 patients with locally advanced cervical cancer underwent treatment. Sixty-seven patients had positive pelvic lymph nodes, of which 53.7% received a nodal boost. Five-year recurrence free survival was 48.6% with vs. 64.5% without nodal boost (*P* = 0.169) and 5-year overall survival in those with positive pelvic lymph nodes was 74.3% with vs. 80.6% without nodal boost (*P* = 0.143). There was no significant difference in toxicity with nodal boost.

**Conclusions:**

EBRT boost to pelvic lymph nodes does not reduce recurrence or improve survival in locally advanced cervical cancer with lymph node involvement at diagnosis.

## Background

Cervical cancer is 4th most common cancer in females worldwide [1]. However, despite advances in treatment modalities and implementation of screening programs, it is still ranked 8th highest in overall cancer mortality rate in Singapore [2]. Current recommendations for locally advanced cervical cancer include pelvic external beam radiotherapy (EBRT) with concurrent cisplatin-containing chemotherapy regimen combined with intra-cavitary brachytherapy [3]. Involvement of pelvic lymph nodes is known to be one of the most important prognostic factors in cervical cancer [4]. In the recently revised FIGO staging, patients with positive pelvic lymph nodes have been included and classified as stage IIIC1 and those who also had positive para-aortic nodes were classified as stage IIIC2 [5]. Prior to this, FIGO staging did not formally classify lymph node involvement and to date there is no standardized guideline on external beam radiation boost to pelvic lymph nodes. However, previous retrospective studies have suggested some benefit of applying EBRT boost to pelvic lymph nodes as recurrence in pelvic lymph nodes is not uncommon following initial definite radiotherapy [6–9]. This study aims to assess the benefit of external beam radiation boost to pelvic lymph nodes in reducing local and distant recurrence rates, improving overall survival as well as its effect on radiotherapy associated toxicities.

## Methods

A retrospective review of medical records of all patients who have undergone radical intent treatment with radiotherapy for locally advanced cervical cancer at our institution between 2008 to 2015 was performed following approval from the National Healthcare Group Domain Specific Review Board, Singapore. None of the patients included in this study had radical hysterectomy or pelvic lymph node dissection. Lymph nodes > 10 mm in diameter were interpreted as enlarged [10, 11]. Radiologically suspicious nodes (lymph nodes < 10 mm with round, irregular shape, or ill-defined edge, occurring as clusters or enhances with intravenous contrast) on computed tomography (CT) scan or magnetic resonance imaging (MRI) scan, and positron emission tomography (PET) avid nodes with a maximum standardized uptake value (SUVmax) > 2.5 were also considered to be involved.

### Radiotherapy

The dose of EBRT ranged from 45 Gy to 50.4 Gy in 1.8 Gy daily fractions given over 25 to 28 fractions with 10 MV photons, 5 days a week. In patients receiving additional external beam radiation boost to pelvic lymph nodes, the dose ranged from 3.6 Gy to 19.8 Gy.

Following CT simulation and planning, radiotherapy treatment was carried out using a 4-field box, 3-dimensional conformal technique. All patients had to adhere to a bladder and bowel preparation protocol for the CT simulation and during the whole course of treatment. The clinical target volume (CTV) included the gross disease, cervix, proximal half of the vagina, parametrial tissues, internal and external iliac lymph node region, and the caudal part of the common iliac lymph node chain (up to the L5-S1 vertebrae junction). Treatment with an extended field (which included the para-aortic lymph nodes with the superior border at the level of the T11-T12 or T12-L1 vertebral junction) was administered to patients with clinically involved para-aortic or common iliac lymph nodes seen on CT, MRI, or PET-CT scans. The inguinal nodes were treated in patients with middle or lower vaginal involvement. In pelvic lymph nodes receiving nodal boost, the margins were 0.5 cm to the CTV and 0.5 to 1 cm to the planning target volume. Prescription of the radiation dose was based on the planning target volume and specified at the isocentre, with homogeneity requirements according to recommendations by the International Commission on Radiation Units and Measurements (ICRU-50). The beam arrangement comprised of a 4-field plan with an anterior-posterior beam arrangement and two lateral beams with multileaf collimators used as shielding.

All patients subsequently underwent image guided high dose rate intracavitary brachytherapy with iridium-192 source.

### Chemotherapy

Patients receiving concurrent chemotherapy were given a standard dose of weekly cisplatin 40 mg/m^2^.

#### Post-treatment follow-up

Following completion of radiotherapy treatment, patients were followed-up every week initially for the first 2 weeks, then 4 weeks later, and subsequently every 3 months for the first year, every 6 months for the 2nd year and annually thereafter. Patients were also regularly reviewed by other disciplines, including gynecologists and medical oncologists. Surveillance for recurrence consisted of history taking and clinical assessment including pelvic examination at every visit and re-assessment CT thorax, abdomen and pelvis with biopsy in the event that a recurrence is suspected. Patients were also assessed for toxicities during each visit using the Common Terminology Criteria for Adverse Events (CTCAE) version 4.0 and managed as appropriate.

### Statistical analysis

Overall survival (OS) was defined as time to death due to any cause and recurrence was based on diagnosis of first recurrence following completion of treatment. Estimation of OS and time to recurrence was derived using Kaplan-Meir analysis. Comparison of outcome was calculated using the Chi square test.

## Results

A total of 139 patients were treated for cervical cancer from 2008 to 2015. At diagnosis, all patients were staged using the International Federation of Gynaecology and Obstetrics (FIGO) 2009 staging. The median follow-up was 25 months (range 1 to 106 months).

Sixty-two patients (44.6%) had no involvement of pelvic lymph nodes, 67 patients (48.2%) had lymph nodes that were enlarged (> 10 mm), radiologically suspicious or had a PET SUVmax > 2.5. Lymph node status was unclear in 10 patients. Squamous cell carcinoma (SCC) histology was most common in 76.2% (*n* = 106) of all patients. In 67 patients with positive pelvic nodes, 79.1% (*n* = 53) had SCC and in 62 patients with negative pelvic nodes, 85.5% (*n* = 53) had SCC (Table 1).

**Table 1:** Patient characteristics

		Pelvic LN negative	Pelvic LN positive	
		(*n* = 62)	(*n* = 67)	
			Nodal boost	No nodal boost
			(*n* = 36)	(*n* = 31)
Age	Median (years)	67	56	56
	Range (years)	36 to 95	33 to 85	37 to 82
Histology	SCC	53	28	25
	Adenocarcinoma	6	4	3
	Adenosquamous	3	2	1
	Small cell	0	1	0
	Mucinous	0	0	1
	Not available	0	1	1
FIGO stage*	IA2	1	0	0
	IB1	8	5	2
	IB2	8	2	0
	IIA	13	5	1
	IIB	15	9	17
	IIIA	0	2	2
	IIIB	16	13	9
	IVA	1	0	0
LN size	Median (mm)	NA	17	15
	Range (mm)	NA	7.7 to 38	10 to 27

*(Abbreviations: SCC* squamous cell carcinoma*, LN* lymph nodes. * International Federation of Gynaecology and Obstetrics (FIGO) 2009 staging.)

Out of 67 pelvic lymph node positive patients, approximately half (53.7%; *n* = 36) had received a boost to the pelvic lymph nodes and 46.3% (n = 31) did not receive boost to the pelvic lymph nodes. The mean size of positive pelvic lymph nodes that received boost was 19.6 mm and the mean size of positive pelvic lymph nodes that did not receive boost was 16.0 mm. The median boost dose was 9 Gy (range of 3.6 Gy to 19.8 Gy) (Table 2). Out of 67 patients who had positive pelvic lymph nodes, 9 patients also had positive para-aortic lymph nodes at diagnosis and received extended field RT covering the involved para-aortic nodes as per our department protocol.

**Table 2:** Treatment characteristics of patients with positive pelvic lymph nodes

	Nodal boost (*n* = 36)	No Nodal boost (*n* = 31)
Concurrent chemotherapy	30	30
Boost technique		
IMRT	6	–
3D Conformal	16	–
AP/PA	11	–
Unknown	3	–
EQD2 (EBRT)		
Mean	57.3 Gy	49.6 Gy
Median	58.4 Gy (Range 49.6 to 69.0 Gy)	50.63 Gy (Range 42.5 to 68.2 Gy)

Concurrent cisplatin-based chemotherapy was given in 84.1% (*n* = 117) of all patients. In 67 patients with positive pelvic nodes, 89.6% (*n* = 60) received concurrent chemotherapy and in 62 patients with negative pelvic nodes, 75.8% (*n* = 47) received concurrent chemotherapy. In 36 patients with positive pelvic nodes who received nodal boost, 83.3% (*n* = 30) received concurrent chemotherapy and in 31 patients with positive pelvic nodes who had no nodal boost, 96.8% (*n* = 30) received concurrent chemotherapy (Table 2).

Of the 67 patients with positive pelvic lymph nodes, 4.5% (n = 3) had local recurrence only, 17.9% (*n* = 12) had distant metastases only, and 17.9% (n = 12) had both local and distant recurrence. Overall, 40.3% (*n* = 27) of patients with positive pelvic lymph nodes developed recurrence (local, distant or both) regardless of whether they received pelvic nodal boost. Comparison of recurrence rates in patients with positive pelvic lymph nodes who received nodal boost and those who did not receive nodal boost can be found on Table 3. Nine patients had positive para-aortic nodes and positive pelvic lymph nodes, of which the majority (77.8%, *n* = 7, received pelvic nodal boost and all received extended field radiotherapy covering the para-aortic nodes). In 27 patients with positive pelvic lymph nodes who developed recurrence, 59.3% (*n* = 16) had pelvic LN size > 15 mm, 25.9% (*n* = 7) had suspicious features on radiological imaging, and 14.8% (*n* = 4) had pelvic LN size > 10 mm but less than 15 mm (Table 4).

**Table 3:** Outcome of treatment with or without pelvic nodal boost in lymph node (LN) positive patients

	Pelvic LN positive		
	All (*n* = 67)	Boost to pelvic lymph nodes (*n* = 36)	No boost to pelvic lymph nodes (*n* = 31)
Recurrence	27 (40.3%)	16 (44.5%)	11 (35.4%)
Local only	3 (4.5%)	2 (5.6%)	1 (3.2%)
Distant only	12 (17.9%)	8 (22.2%)	5 (16.1%)
Local and distant	12 (17.9%)	6 (16.7%)	5 (16.1%)
No recurrence	40 (59.7%)	20 (55.5%)	20 (64.6%)
	Pelvic LN and para-aortic LN positive		
	All (*n* = 9)	Boost to pelvic lymph nodes (*n* = 7)	No boost to pelvic lymph nodes (*n* = 2)
Recurrence	6 (66.7%)	4 (57.1%)	2 (100%)
Local only	0	0	0
Distant only	2 (22.2%)	1 (14.2%)	1 (50%)
Local and distant	4 (44.4%)	3 (42.9%)	1 (50%)
No recurrence	3 (33.3%)	3 (42.9%)	0

**Table 4:** Recurrence rate in patients with positive pelvic lymph nodes (LN) based on LN size

Size of pelvic LN	Recurrence (*n* = 27)
> 15 mm	16 (59.3%)
10 mm > LN size < 15 mm	4 (14.8%)
Suspicious features	7 (25.9%)

**Fig. 1:**
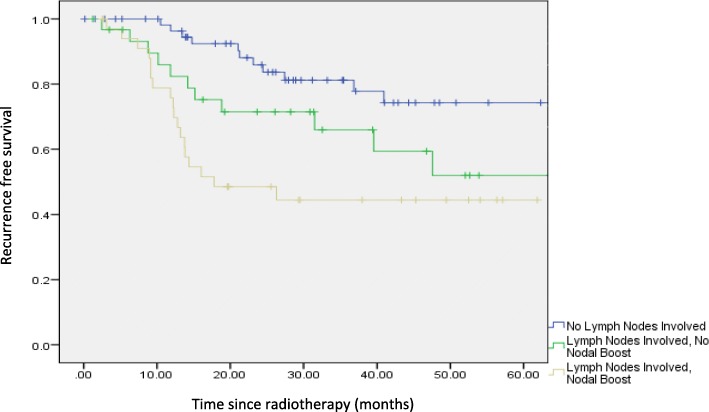
Recurrence free survival in patients with no lymph nodes involved, with positive pelvic lymph nodes with and without nodal boost

**Fig. 2:**
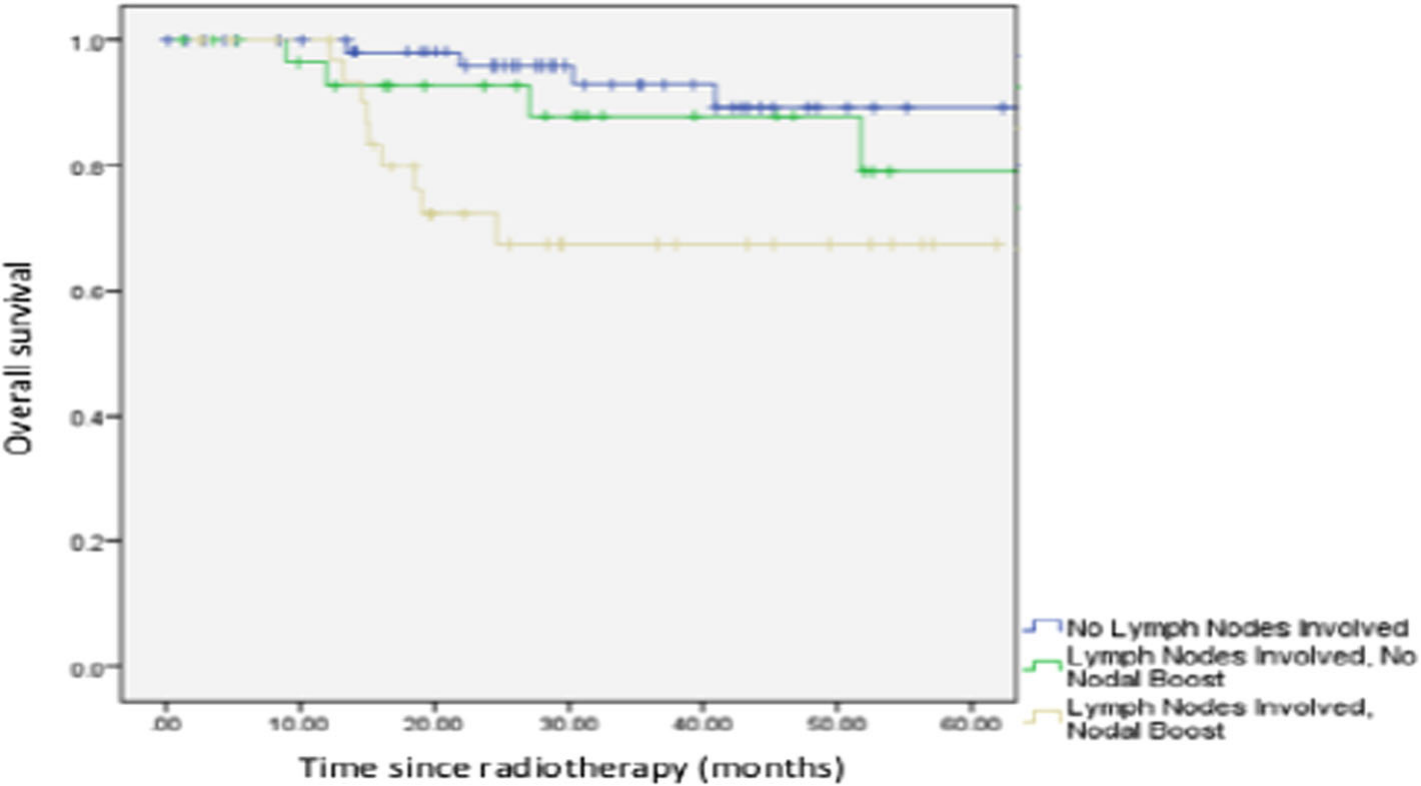
Overall survival in patients with no lymph nodes involved, with positive pelvic lymph with boost, and positive pelvic lymph nodes without nodal boost

The 5-year recurrence-free survival (RFS) was 82% for pelvic lymph node negative patients; 64.5% for pelvic lymph node positive patients with no nodal boost; and 48.6% for pelvic lymph node positive patients with nodal boost (Fig. 1). The 5-year overall survival (OS) rate was 93.4% for pelvic lymph node negative patients; 80.6% for positive pelvic lymph nodes with no nodal boost; and 74.3% for positive pelvic lymph nodes with nodal boost (Fig. 2). There was no significant difference in the development of both early and late toxicities including proctitis, cystitis and fracture of femoral head (Table 5).

**Table 5:** Toxicity

	Toxicity	Nodal boost (*n* = 36)	No Nodal boost (*n* = 31)
Proctitis	G1	1	0
	G2	5	6
Cystitis	G2	1	2
	G3	2	1
Femoral head	Fracture	1	1

Graded according to CTCAE version 4.0 (Proctitis Grade 1: Rectal discomfort, no intervention, Grade 2: Rectal symptoms, intervention indicated. Cystitis Grade 2: Moderate haematuria, Grade 3: Gross haematuria)

## Discussion

In this study, we identified 67 patients with positive pelvic lymph nodes, of which 53.7% (*n* = 36) received nodal boost to pelvic lymph node and 46.3% (n = 31) did not. We found that administering nodal boost to pelvic lymph nodes did not significantly improve 5-year RFS (48.6% with nodal boost vs. 64.5% without nodal boost, *P* = 0.169). There was also no significant difference in OS in locally advanced cervical cancer with positive pelvic lymph nodes (74.3% with nodal boost vs. 80.6% without nodal boost, *P* = 0.143). In fact, patients with positive pelvic lymph nodes who did not receive a nodal boost appeared to have fewer recurrences and better OS, however these were not statistically significant. In both groups of patients, recurrence tends to occur as distant metastases and a boost did not decrease local nodal recurrence. Recurrence rate in patients with positive pelvic lymph node who received concurrent chemoradiotherapy were comparable in those with nodal boost (33.3%, *n* = 12) and those without nodal boost (35.5%, *n* = 11).

There is a need for pelvic lymph node status to be uniformly assessed at the time of diagnosis and subsequent follow-up. Grigsby et al. [12] compared CT scan with fluorodeoxyglucose (FDG)-PET scan for lymph node staging in patients with cervical cancer and found FDG-PET to be superior to CT scan in detecting abnormal lymph nodes. FDG-PET would also be invaluable in detecting distant metastases which is significantly increased in lymph node positive patients [4, 7, 8, 13]. However, cost of FDG-PET scan may be a limiting factor in clinical practice. In our study, only 18.7% (*n* = 26) of all patients had PET scan as part of their staging mainly due to cost limitation.

The significance of pelvic lymph node involvement in indicating future recurrence has led to studies attempting to assess the benefit of a nodal boost to pelvic lymph nodes. However, results have been conflicting. In a retrospective study of 174 patients with locally advanced cervical cancer by Ariga et al. [7], 33% (*n* = 58) had positive pelvic lymph nodes, of which the majority (91%) were given nodal boost and comparisons were then made against those who had negative pelvic nodes with no nodal boost. They demonstrated significant improvement in 5-year OS and disease-free survival rate for patients with positive and negative nodes (73% vs. 92%, *P* = 0.001; 58% vs. 84%, *P* < 0.001, respectively). Overall, they reported benefit of nodal boost in patients with positive pelvic lymph nodes as compared to those with negative pelvic lymph nodes who had no boost and suggested that nodal boost could be an alternative to surgical debulking of pelvic lymph nodes. In a small retrospective study involving 32 patients with pelvic lymph node positive cervical cancer who were treated with EBRT without boost irradiation to the pelvic lymph nodes, Yoshizawa et al. [14] reported that only 2 patients had failure in the pre-existing lymph nodes and that 95% of those who progressed did so with distant metastases. They therefore suggested that pelvic lymph node boost may not be necessary especially in view of the potential toxicities.

To the best of our knowledge, this is the first study that directly compares the outcome of EBRT boost to pelvic lymph nodes in patients with positive pelvic lymph nodes in locally advanced cervical cancer. While it may seem intuitive that a higher radiotherapy dose to gross disease should result in a better outcome, this is not evident in our study. One reason could be that cervical cancer is inherently radiosensitive and that EBRT with some dose contribution from the brachytherapy provides sufficient dose to achieve a cure. We also note that in our cohort, a slightly higher proportion of patients with positive pelvic lymph nodes who had no nodal boost received concurrent chemoradiotherapy (96.8% vs. 83.3%). Although the discrepancy was small and not statistically significant, future study design could perhaps aim to eliminate this discrepancy altogether so outcome can be more accurate given chemotherapy’s role as a radiosensitizer.

In our cohort of patients with positive pelvic lymph nodes, approximately one third of patients developed recurrence despite definitive treatment. As resistance to radiotherapy have previously been suggested as a factor of treatment failure, predictive biomarkers to identify radiation resistance cells will be invaluable in selecting patients who will respond well to radiation while those with more resistance cells can be considered for additional treatment to improve treatment outcome. Unnecessary toxicities can also be spared in select groups [15].

## Conclusion

Our results show that EBRT boost to pelvic lymph nodes did not improve OS nor reduce rate of local recurrence in patients with locally advanced cervical cancer. However, prospective studies are needed to establish evidence-based guidelines on EBRT boost to pelvic lymph nodes in locally advanced cervical cancer.

## Abbreviations

CT: Computed tomography
CTCAE: Common terminology criteria for adverse events
CTV: Clinical target volume
EBRT: External beam radiation therapy
FDG: Fluorodeoxyglucose
FIGO: International Federation of Gynaecology and Obstetrics
ICRU-50: International Commission on Radiation Units and Measurements report 50
LN: lymph node
MRI: Magnetic resonance imaging
OS: Overall survival
PET: Positron emission tomography
RFS: Recurrence free survival
SUVmax: Maximum standardized uptake value
